# Pulsed Fluidization of Nanosilica: Rigorous Evaluation of the Efficacy of Pulsation Frequency

**DOI:** 10.3390/nano12132158

**Published:** 2022-06-23

**Authors:** Mohammad Asif, Ebrahim H. Al-Ghurabi, Amanullah Fatehmulla

**Affiliations:** 1 Department of Chemical Engineering, King Saud University, P.O. Box 800, Riyadh 11421, Saudi Arabia; ealghurabi@ksu.edu.sa; 2 Department of Physics and Astronomy, College of Science, King Saud University, P.O. Box 2455, Riyadh 11451, Saudi Arabia; aman@ksu.edu.sa

**Keywords:** nanosilica, fluidization, pulsed flow, pulsation frequency, assisted fluidization, efficacy, bed dynamics

## Abstract

Assisted fluidization techniques can significantly improve the hydrodynamics of difficult- to-fluidize solids. Among these techniques, the pulsed flow strategy is highly promising owing to its cost-effectiveness and amenability to implementation for largescale processing. Using commercial-grade, highly porous nanosilica that shows strong agglomeration behavior, we implemented the pulsed flow with square-wave pulsation schemes of 0.05, 0.10, and 0.25 Hz frequencies, and compared their effectiveness in each case. Besides the conventional approach of assessing their efficacy using the pressure drop data, we have proposed a new approach in this work that consists of computing the power of the overall pressure drop transient signals. Using the theoretical value, i.e., the effective bed weight per unit area as a reference, the percentage increase in the power was 27 ± 4, 71 ± 5, and 128 ± 4, respectively, for 0.05, 0.10, and 0.25 Hz pulsation frequencies. In fact, the average pressure drop values were substantially higher when the partial bed collapse occurred between successive pulsations when compared with the case of low-frequency pulsations. The pulsation frequency also affected the evolution of local bed dynamics in various bed regions during the expansion and collapse of the bed. Moreover, the local and global pressure transients have shown interesting mutual correlations which were otherwise not evident from their individual transient profiles.

## 1. Introduction

Notwithstanding inherent advantages associated with the use of fine and ultrafine powders, their processing remains hugely challenging. This was pointed out long ago by Geldart [1], who examined the effect of the physical properties of the solid particles on their gas-phase fluidization behavior. For particles with a size smaller than 30 µm, the solids, when contacted with an upward gas flow, show cohesive and non-homogeneous fluidization behavior with poor contact and insufficient mixing between the two phases. This leads to poor transport of heat and mass that could ultimately compromise the effectiveness of the process.

A commonly used strategy to improve the fluidization hydrodynamics of difficult-to-fluidize cohesive solids has been the use of assisted fluidization techniques [2]. These techniques mostly require the input of additional extra energy to the system mainly to counteract the interparticle forces, thereby enhancing the fluidization behavior. For instance, subjecting the fluidized bed to mechanical vibration leads to a greater bed homogeneity and reduced minimum fluidization velocity (U_mf_) in addition to uniform product quality [3,4,5,6,7,8,9,10,11,12]. Given that the transition of the fixed bed to the fluidized bed mode of gas–solid contact occurs at the U_mf_, it is therefore considered a critical fluidization parameter. Similarly, a pulsation of the inlet flow promotes deagglomeration, reduces the U_mf_, and suppresses the hysteresis phenomenon [13,14,15,16,17,18,19,20,21,22,23,24]. Another assisted fluidization strategy widely reported in the literature utilizes the acoustics perturbations generated from a sound source. When the acoustic frequency matches the natural frequency of the system, resonance occurs, which leads to the development of standing waves, thereby causing high-intensity disturbances in the pressure transients. This lowers the U_mf_ and improves the quality of fluidization [25,26,27,28,29]. Mixing with inert particles of appropriate physical properties has also been reported as a strategy to enhance the fluidization hydrodynamics of difficult-to-fluidize particles [30,31,32,33]. In some cases, a combination of two different assisted fluidization techniques has been utilized to improve the fluidization quality and suppress the size segregation of the bed material along the height [20,34,35,36].

For largescale gas–solid processing, the efficacy of the energy augmentation using the assisted fluidization technique is of critical importance for the economic feasibility of the process. In this context, the pulsed flow assisted fluidization technique stands out on two main counts. First, unlike mechanical vibrations and acoustic perturbations, additional energy input and its associated capital and operating costs are not required. Second, the existing process units can be easily and economically retrofitted to introduce flow pulsations.

Pulsed flow introduces intense pressure fluctuations, which cause additional dissipation of energy and can therefore help to improve fluidization hydrodynamics. Simple flow pulsation strategy, e.g., square-wave flow pulsations, can be implemented by regularly interrupting the inlet fluid flow. Once the flow is stopped, the collapse process begins. As a result of regular expansion and collapse of the bed due to pulsed flow, the solid phase mostly remains in a state of vigorous perpetual motion, thus leading to additional frictional losses, which are otherwise not possible with conventional fluidization.

Pulsation frequency can be adjusted to cause either partial or total collapse of the bed between two successive pulsation events. Further lowering the pulsations frequency beyond that required for complete collapse would provide additional time for the adjustment of solid particles before the next pulsation event [14,17,24].

The efficacy of assisted fluidization techniques has mostly been assessed in terms of the U_mf_ reduction and the fluidization index, which is a good indication of the correspondence between the overall pressure drop and the bed’s effective weight. While a qualitative indication of the quality of the fluidization using this approach is possible, a precise evaluation of the power augmentation as a result of applying the assisted fluidization technique is nonetheless not feasible. In this study, we therefore set out to make a rigorous estimation of the energy dissipation per unit time from the pressure transients as a tool to discern the efficacy of the pulsed flow with different pulsation frequencies in a highly porous bed of nanosilica. The proposed approach, although presented here in the context of pulsed flow, can be extended to other assisted fluidization techniques as well. We have considered three different square-wave frequencies: 0.05, 0.10, and 0.25 Hz. While low frequency yields sufficient time between two consecutive pulsation events, higher frequencies, i.e., 0.10 Hz and 0.25 Hz, respectively, allow complete and partial collapse of the bed between successive pulsations. Following the conventional approach, we examined the effect of the pulsation frequency on the average values of the local as well as the overall pressure drop as a function of the gas flow. Next, the correlation between the local and global dynamics of different bed regions in response to the pulsations has been analyzed. Finally, the energy dissipation per unit time has been evaluated from the overall pressure signal to evaluate the effect of the pulsed flow frequency on the power input.

## 2. Experimental

**Figure 1:**
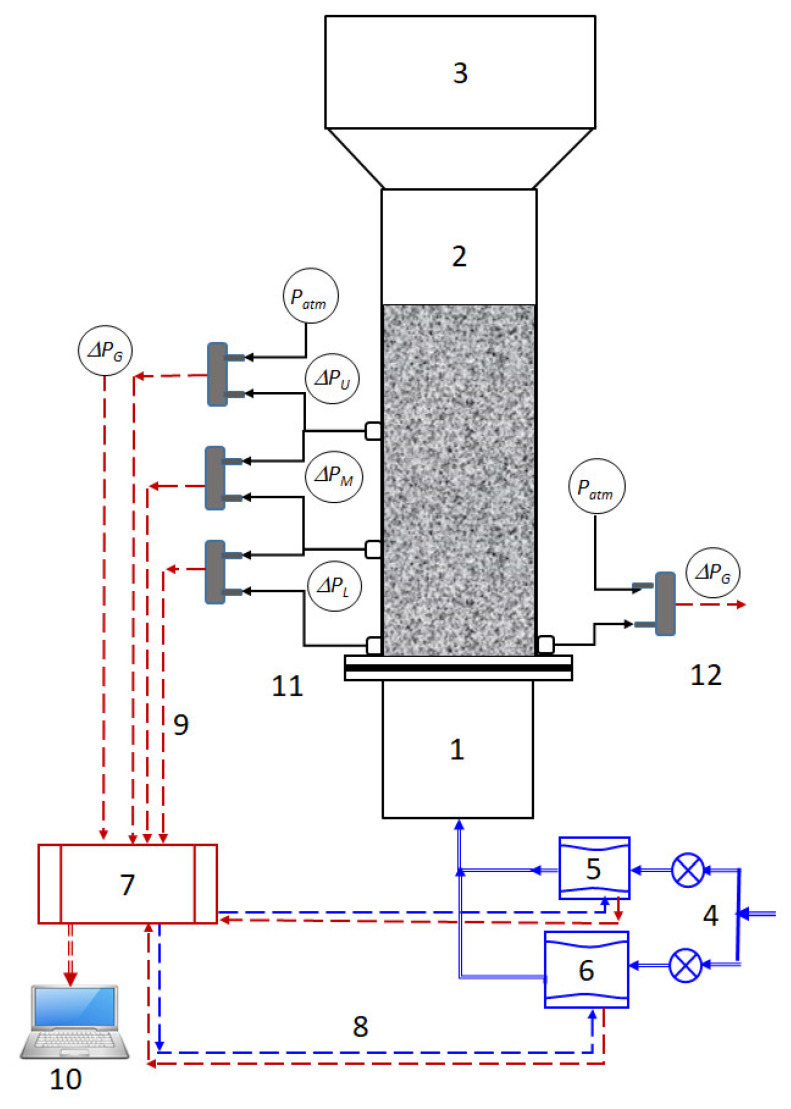
Experimental setup schematic. (1) Wind-box; (2) Test section; (3) Disengagement section; (4) Compressed air supply; (5) Mass flow controller (low flow); (6) Mass flow controller (high flow); (7) Data acquisition system (DAQ); (8) Analog output (AO) signals (blue broken lines) (9) Analog input (AI) signals (red broken lines); (10) Laptop with LabVIEW software; (11) Pressure taps for upper ($∆P_{U}$), middle ($∆P_{M}$ ), and lower ($∆P_{L}$ ) pressure drops; (12) Pressure taps for global pressure drop ($∆P_{G}$ ).

The schematic of the experimental set used in this study is shown in Figure 1. The test section was a 1.5 m long transparent Perspex column with an internal diameter of 70 mm, which was preceded by a perforated plate distributor and a 0.5 m long plenum chamber. The fractional open area of the distributor was kept low to ensure uniform gas distribution by eliminating the dead zones [37,38].

Several sensitive pressure transducers with appropriate ranges were used for monitoring the bed transients. Local bed dynamics were monitored in the lower ($∆P_{L}$), middle ($∆P_{M}$), and upper ($∆P_{U}$) regions of the fluidized bed. Overall bed transients ($∆P_{G}$) were also monitored with a pressure transducer of relatively larger range as compared to the ones used for monitoring local transients. The bottom tap of this transducer was located close to the distributor, whereas the upper tap was kept open to the atmosphere as shown in Figure 1. The transient pressure drop data were recorded at a frequency of 100 Hz with an 18-bit data acquisition system (DAQ) (Model: USB-6289, National Instruments, Austin, TX, USA) controlled using LabVIEW 2019 software obtained from National Instruments, Austin, TX, USA.

We used commercial-grade hydrophilic nanosilica (Aerosil 200, Evonik GmbH, Wolfgang, Germany), which is widely used in bulk quantities in various applications, e.g., rubber, plastics, concrete, agriculture, and cosmetics, with an estimated 3.3 million tons of global consumption in 2015 [39]. Due to multi-level agglomeration caused by interparticle forces, the particle size distribution of its sieved sample ranged from 2 to 200 μm, which was three orders of magnitude greater than the actual size of the nanoparticle [14]. The bulk density was 44 kg/m^3^ with an overall bed void fraction of approximately 0.98.

Compressed air was used as the fluidizing gas. Its flow was controlled by using two different electronic mass flow controllers. Both flow controllers were connected to the DAQ to generate pulsed flow. Square-wave flow pulsation schemes were implemented for three different frequencies: 0.25, 0.10, and 0.05 Hz. For 0.25 Hz, the gas flow was maintained for two seconds followed by another two seconds of pause, thus completing one cycle. Likewise, gas flow durations were kept for five seconds and ten seconds followed by a pause of Perspex column the same duration for the 0.10 and 0.05 Hz cases, respectively. These frequencies were carefully chosen to cover the full spectrum of the bed collapse such that 0.25 Hz flow pulsation allowed only a partial collapse of the bed before the next pulsation event. However, 0.10 Hz was just sufficient to ensure the complete bed collapse in most cases before the start of the next pulsation, whereas a 0.05 Hz flow pulsation ensured a complete bed collapse and its settlement before the next flow pulse.

**Figure 2:**
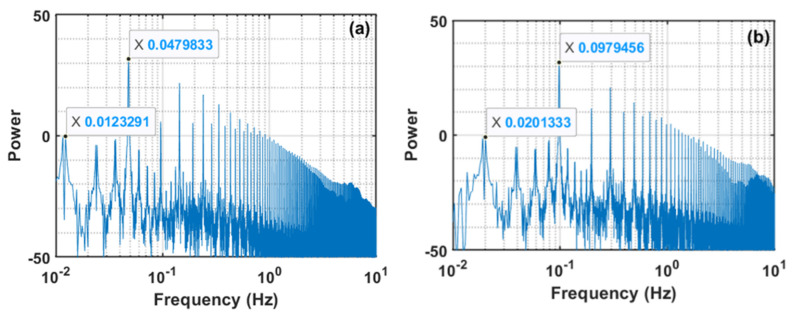

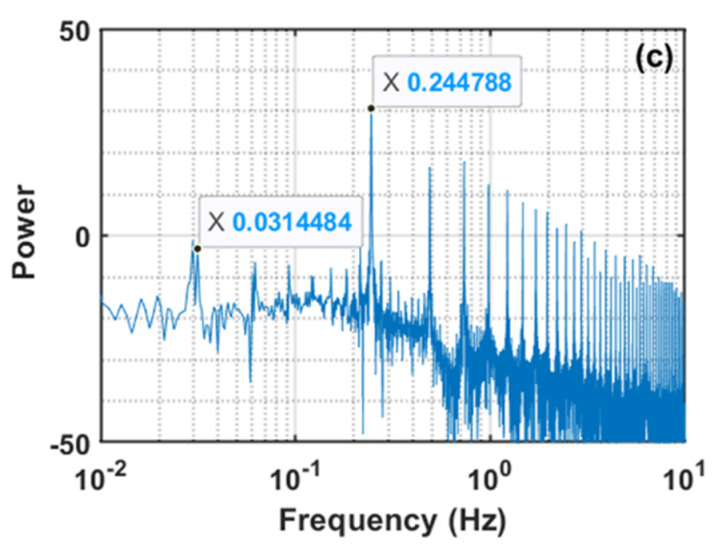
Power spectra (in dB) of the velocity data for (**a**) 0.05 Hz, (**b**) 0.10 Hz, (**c**) 0.25 Hz.

Our experimental strategy consisted of using 36 velocities such that 18 different flows were conducted out by gradually increasing the flow whereas the next 18 flows consisted of gradually decreasing the flow, thereby completing both the fluidization and defluidization cycles of an experimental run. At a fixed flow of the fluidizing gas, four complete pulses, each of 20 s duration, were carried out for a 0.05 Hz pulsed flow. Similarly, five complete pulses, each of 10 s duration, were considered for 0.1 Hz pulsations for each individual flow. In the case of 0.025 Hz, however, we considered eight pulses, each of 4 s duration, for a given flowrate. These aspects of our experiments are highlighted in Figure 2, which shows the power spectra of the velocity data for a complete experimental run. A prominent peak in Figure 2a at 0.048 Hz corresponds to the pulsation frequency, while another peak at 0.0124 Hz corresponds to the time duration of 80 s for which the velocity was held constant, thereby yielding four identical flow pulses with a time period of 20 s. In Figure 2b, we likewise note a prominent peak at 0.098 Hz, indicating the frequency of flow pulsation. Another peak at 0.020 Hz corresponds to the flow changes introduced after 50 s. In the case of 0.25 Hz, flow pulsation is depicted in Figure 2c with velocity step changes introduced at an interval of 32 s, where each pulse lasted only four seconds.

**Figure 3:**
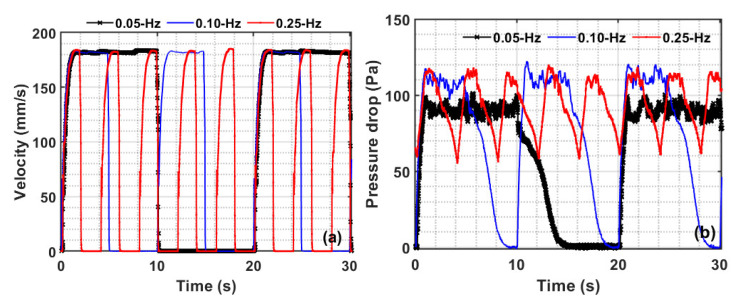
Pulsed flow transients for (**a**) the velocity and (**b**) the upper region pressure drop for different frequencies of the pulsed flow.

A 30 s snapshot of the velocity and pressure drop transients in real time is shown in Figure 3. As seen in Figure 3a, the flow lasted for 10 s and was interrupted for the next 10 s for a 0.05 Hz pulsed flow, which was reduced to five seconds for the 0.1 Hz and further reduced to 2 s for the 0.25 Hz pulsed flow. From the overall dynamics shown in Figure 3b it is obvious that the collapse process for 0.05 Hz is complete, with the bed coming to a complete rest with subsequent adjustment, if any, before the next pulse of flow is introduced. In the case of 0.1 Hz, however, the flow interruption duration was just sufficient for the total bed collapse to complete between two successive pulsation events. As the pulsation frequency was raised to 0.25 Hz, there was a notable change in the bed dynamics. The overall pressure hardly dropped to 50 Pa before the next flow pulse was introduced. This means that the 0.25 Hz flow pulsation frequency allowed only a partial bed collapse. As a result of different dynamics seen with different frequencies, a significant difference in the bed hydrodynamics is expected.

In view of the square-wave flow pulsations, the following equations were used for computing the mean values of the velocities ($U_{0}$) and the pressure drops (∆P),

(1) $$U_{0}=\frac{1}{N}\overset{N}{\underset{i=1}{\sum }}\overset{n_{2}}{\underset{j=n_{1}}{\sum }}\frac{U_{0ij}}{\left(n_{2}-n_{1}+1\right)},$$

(2) $$∆P=\frac{1}{N}\overset{N}{\underset{i=1}{\sum }}\overset{n_{2}}{\underset{j=n_{1}}{\sum }}\frac{∆p_{ij}}{\left(n_{2}-n_{1}+1\right)},$$

where $U_{0ij}$ and $∆p_{ij}$ are the *j*^th^ data points of the *i*^th^ pulse for the velocity and the pressure drop, respectively. For 0.25 Hz pulsations as an example, there were eight pulses (*N* = 8), each of four-second duration, with data acquired with a sampling frequency of 100 Hz, thereby yielding 400 data points. The part of the pulse with zero flow was not considered. For the remaining part of the pulse comprised of 200 data points, its initial rise time and the latter part of the pulse were also discarded. Therefore, we used $n_{1}=60$ and $n_{2}=160$ for the 0.25 Hz pulsed flow.

## 3. Results and Discussion

In the following, the effect of the gas flow on the local and overall pressure drop is first examined for various pulsation frequencies. The correlation between the dynamics of different bed regions is highlighted next. Finally, the influence of the pulsation frequency on the energy dissipation per unit time is discussed.

### 3.1. Dependence of the Local Pressure Drop on Gas Velocity

**Figure 4:**
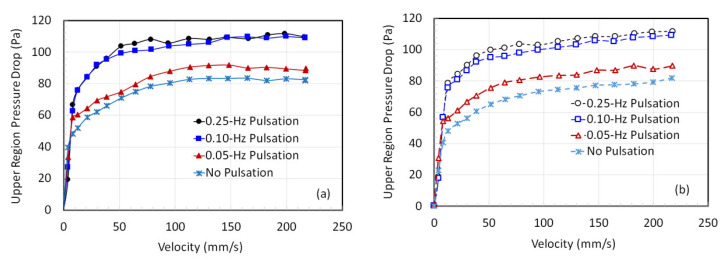
Local pressure drop variation with the velocity in the upper region with (**a**) increasing velocity and (**b**) decreasing velocity.

During the square-wave pulsed flow, the flow stoppage initiated the collapse, thereby causing a simultaneous decline in the pressure drop as well. However, for the evaluation of the average pressure drop, the pressure drop magnitudes for the fully developed were considered [24]. The variation in the local pressure drop in the upper region of the fluidized bed with the velocity is shown in Figure 4 for various pulsed flows. The case of conventional fluidization with no pulsation is also depicted in the figure for the sake of comparison. The 0.25 Hz and 0.1 Hz cases show close agreement with much greater pressure drop as compared to 0.05 Hz, which was still slightly higher than the conventional fluidized bed. This behavior is seen for both when the flow was gradually increased (Figure 4a) followed by a gradual decrease (Figure 4b). The steady increase in the pressure drop with the velocity occurs due to the bed expansion, which causes a greater input of bed solids in the upper region. A higher pressure drop indicates a greater expansion of the fluidized bed.

**Figure 5:**
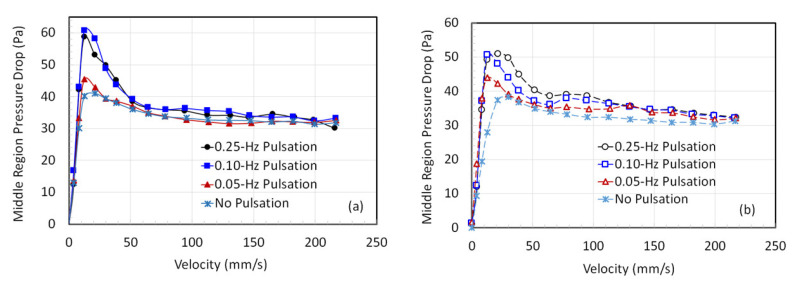
Local pressure drop variation with the velocity in the middle region with (**a**) increasing velocity and (**b**) decreasing velocity.

The pressure drop variation with the flow in the middle region of the fluidized bed is highlighted in Figure 5. The pressure drop at higher velocities, i.e., above 50 mm/s, is comparable for all cases during the fluidization cycle (Figure 5a). Similar behavior is also observed during the defluidization cycle in Figure 5b. At lower velocities in Figure 5a, below the U_mf_, the pressure drop for 0.25 and 0.1 Hz is higher than that for 0.05 Hz. Assuming the applicability of the Ergun equation for flow in fixed beds, a higher pressure drop can be attributed to the smaller size of agglomerates [14].

**Figure 6:**
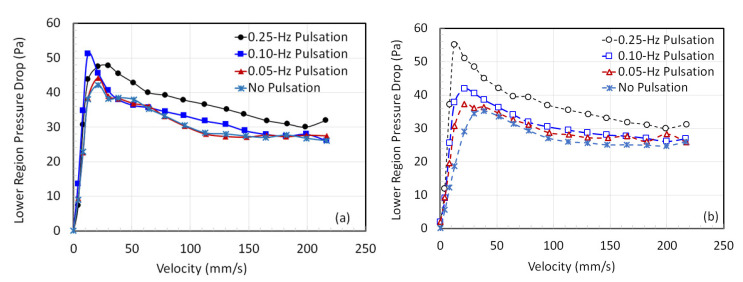
Local pressure drop variation with the velocity in the lower region with (**a**) increasing velocity and (**b**) decreasing velocity.

The pressure drop variation with the velocity in the lower region of the pulsed bed is shown in Figure 6 for both fluidization (Figure 6a) and defluidization (Figure 6b) cycles. The highest frequency pulsed flow in this case differs from all other cases especially during the gradual velocity decrease cycle of the experimental run (Figure 6b). This is caused by the partial collapse of the pulsed bed where the frequent collapse events cause a greater compaction, thus leading to higher frictional losses in the lower region.

**Figure 7:**
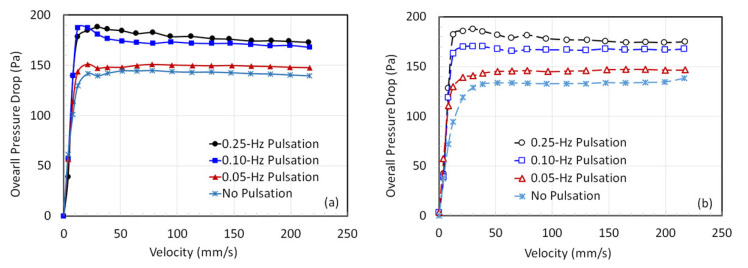
Variation of the total pressure drop with the velocity in pulsed beds with (**a**) increasing velocity and (**b**) decreasing velocity.

Next, in Figure 7, we consider the overall total pressure drop across the fluidized bed. The experimental data of the fluidization (Figure 7a) and defluidization (Figure 7b) cycles show the absence of hysteresis behavior. Moreover, the 0.25 and 0.10 Hz pulsed flows show good agreement with a significantly higher pressure drop than the corresponding 0.05 Hz case, which happens to be marginally higher than the one for the un-pulsed flow (conventional fluidization). A clear difference is seen in the defluidization cycle of the experiment. The 0.05 Hz flow pulsation clearly yields a slightly higher pressure drop than that for the un-pulsed flow. Likewise, 0.25 Hz pulsation causes a higher pressure drop than the one for 0.10 Hz. Note that the effective weight per unit area of the fluidized bed is approximately 149 Pa, whereas we often obtain pressure drop values as high as 185 Pa for the fully fluidized bed with the introduction of flow pulsations. This issue will be discussed in detail in Section 3.3.

A close look at Figure 7b also reveals that pulsation helps lower the U_mf_. However, any clear difference between the 0.1 and 0.25 Hz is hardly noticeable. In fact, earlier studies relied on the evaluation of the agglomerate diameter either from the U_mf_ or the bed collapse data to assess the effect of the flow pulsation [15,16]. Such an approach is based on the validity of a theoretical model capable of describing the experimental data. Therefore, any error in the predictive capability of the model will consequently affect the assessment of the efficacy of the assisted fluidization technique.

**Figure 8:**
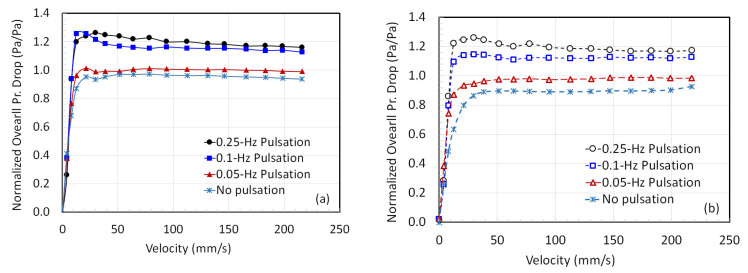
Variation in the normalized pressure drop with the velocity in the pulsed bed with (**a**) increasing velocity and (**b**) decreasing velocity.

We have presented the normalized pressure drop behavior in Figure 8 where the actual experimental pressure drop was divided by the effective bed weight per unit area. In the literature this parameter is sometimes called the fluidization index [33,40,41]. The results shown in the figure are quite revealing, especially during the defluidization leg of the experiment when the velocity was gradually decreased. In the absence of flow pulsation, the fluidization index was almost 0.9. This means that the pressure drop was less than the effective solid weight per unit bed area. When the low frequency flow pulsations of 0.05 Hz were introduced, the fluidization quality improved. The value of the normalized pressure drop was approximately 0.99. At 0.10 Hz, we noted an increase in the normalized pressure drop to a value of approximately 1.12 for most velocities. This is a more than 20% increase in the pressure drop compared to what was obtained without pulsations. Increasing the frequency to 0.25 Hz further increased the normalized pressure drop to approximately 1.2.

The fluidized bed pressure drop is essentially caused by frictional losses, which arise from changes in the kinetic and potential energy, as well as by gas–solid interphase and solid–solid intra-phase drag. During the bed collapse, the potential energy converts into kinetic energy as the solids fall downwards. This causes an increase in the gas–solid interphase drag, leading to greater energy dissipation. In fact, this phenomenon also occurs during the expansion of the bed. Thus, the frequent collapse and expansion of the pulsed bed led to greater energy dissipation as compared to the case of conventional fluidization. As the pulsation frequency increased, greater energy dissipation took place, which was reflected in greater frictional losses. In the present case, 0.25 Hz pulsations caused a substantially greater power augmentation in the pulsed bed as compared to the one for conventional fluidization.

### 3.2. Bed Dynamics and Their Inter-Region Correlation

**Figure 9:**
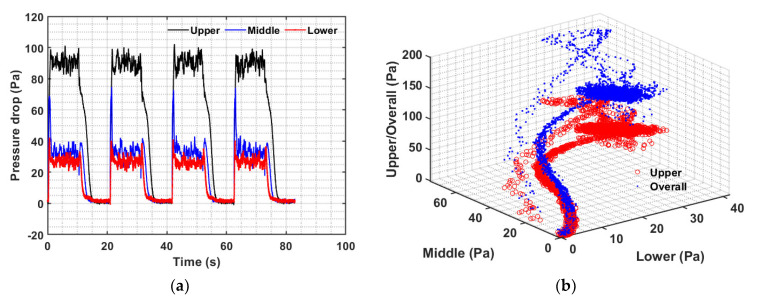
(**a**) Bed transients in different regions of the pulsed fluidized bed and (**b**) inter-region pressure drop data correlation for 0.05 Hz at 182 mm/s.

The dynamics in various bed regions were examined by utilizing the experimental data of the entire spectrum of several pulses at a fixed flow rate. First, let’s consider the case of 0.05 Hz in Figure 9 with 10 s of gas flow followed by another 10 seconds of pause at a velocity of 182 mm/s. There were four complete pulses with a total duration of over 80 seconds, with approximately 8000 pressure-transient data points for each of the three regions in addition to data points for the overall pressure drop transients. The results are shown in Figure 9a. At the beginning of the flow pulse, all regions immediately responded to the change in flow. The vigorous solid motion was captured by the disturbances in the pressure transients. Once the flow was stopped, the bed collapse started. As expected, the effect of the bed fall was felt first in the lower portion, followed by the middle region, and subsequently the upper part.

Because the dynamics in all three bed regions occurred simultaneously, they were therefore correlated. The mutual interdependence regional dynamics are presented in Figure 9b, which also depicts the transients of the overall bed pressure drop. Note that the global dynamics data were recorded using the pressure transducer with its lower port above the distributor and the upper port open to the atmosphere. The highest data concentration is seen in the form of a circular disk. It represents the steady part of the flow pulse where the gas velocity was constant. There are two different branches, with different amounts of data points, seen in the figure; one leads toward the data disk and the other emanates from it. The branch with fewer data represents the bed expansion, while the other branch shows the bed collapse. This is a clear indication that expansion transients are faster than collapse transients.

**Figure 10:**
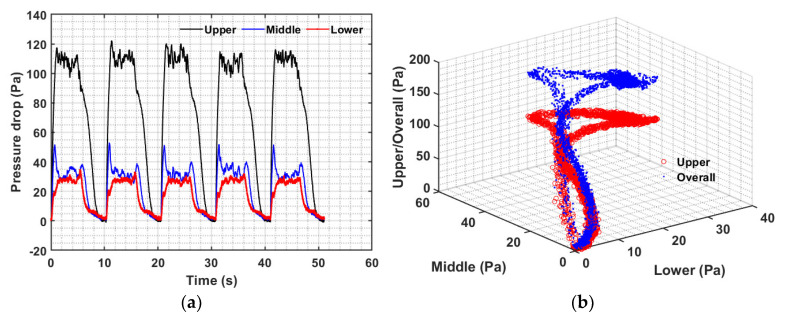
(**a**) Bed transients in different bed regions and (**b**) inter-region pressure drop data correlation for 0.10 Hz at 182 mm/s.

The case of 0.10 Hz pulse with five seconds of gas flow followed immediately by another five seconds of flow interruption at 182 mm/s is considered in Figure 10. There were five complete pulses with a total duration of over 50 seconds, as seen in Figure 10a. There is a substantial consistency in the dynamics of all the five pulses. Expectedly, pressure transients in all the regions immediately responded to the flow change, clearly evident at the start of the flow pulse. The middle region transient immediately peaks, whereas the lower region lags behind. The motion of the solid phase in the bed was once again evident from the disturbances occurring in the pressure transients. The steady part of the pulse was significantly less dense than the previous case of 0.05 Hz pulse. Once the bed collapse begins with the flow interruption, the upper region transients show significant lag compared with the transients of the other two regions, yet the complete bed occurred before the next flow pulse.

The interdependence of the dynamics of all three regions is shown in Figure 10b. The data in the form of the circular disk are significantly smaller than the 0.01 Hz counterpart due to the much shorter time during which the bed is under relatively steady flow conditions. Two different branches, leading to and emanating from the circular data region, are once again clearly visible. Faster expansion transients possess fewer data as compared to the collapse dynamics. The curvature in the expansion branch arose from the faster transients displayed by the middle portion of the bed as compared to the transients of the lower portion.

**Figure 11:**
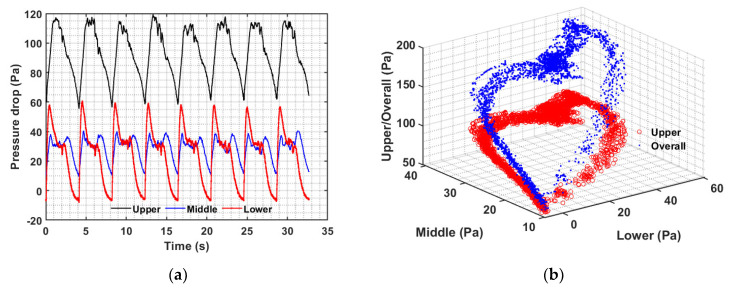
(**a**) Bed transients in different bed regions and (**b**) inter-region pressure drop data correlation for 0.25 Hz at 182 mm/s.

The case of 0.25 Hz flow pulsations is shown in Figure 11 with two seconds of flow followed by the remaining two seconds of flow cessation. There was a total of eight pulses for a fixed velocity with a total duration of 32 seconds. Unlike the previous case of 0.05 Hz and 0.1 Hz, only a partial collapse of the bed occurred such that solids in the upper portion of the pulsed bed remained in a state of perpetual motion. The lowest pressure drop remained approximately 60 Pa, while the peaks were significantly higher than that for 0.05 Hz and slightly higher than that for 0.10 Hz pulsations. The middle region appeared relatively insensitive to pulsations as compared to the other two regions, i.e., the upper and lower regions. Another peak in the middle region pressure transients after the start of the collapse is due to the addition of the solid mass from the upper part. This significantly slows the transients of the middle region. Moreover, the central portion of the bed was never settled during the pulsed flow. Transients in the lower portion were pronounced with a prominent initial peak and a steep drop in pressure with the bed fall. This behavior significantly differed from that seen for the 0.05 and 0.10 Hz pulsed flows.

The transient behavior seen in Figure 10a is also clearly reflected in Figure 11b. The faster transients in the lower bed region now shift the data branch movement counterclockwise toward the disk-shaped data region. Note that the upper region or the overall pressure drop was always above 50 Pa. Similarly, the middle region pressure drop always remained above 10 Pa, while the lower region pressure drop became negative toward the end of the collapse.

### 3.3. Energy Dissipation per Unit Time

**Figure 12:**
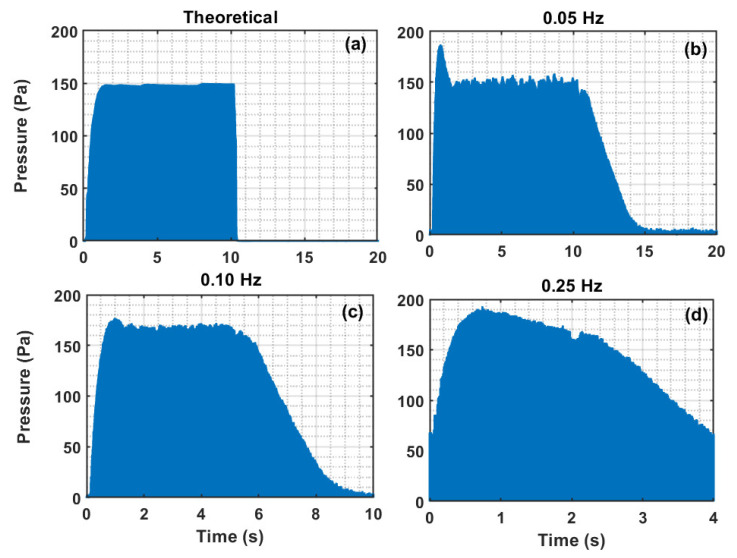
Shaded area used in the evaluation of the integral expression used in Equation (3); (**a**) Theoretical values (effective bed weight) based on 0.05 Hz pulsation, (**b**) 0.05-Hz pulsation, (**c**) 0.10-Hz pulsation, (**d**) 0.25-Hz pulsation.

To investigate the effect of the pulsation on the power augmentation, we have used the commonly suggested approach, which consists of evaluating the square of the signal over time (*T*) as follows:

(3) $$Power=\underset{T\to \infty }{\lim}\frac{1}{T}\overset{T}{\underset{0}{\int }}∆P_{G}^{2}dt,\;$$

where $∆P_{G}$ is the overall pressure drop signal across the pulsed bed. Four different cases are depicted in Figure 12 for the estimation of the integral in Equation (3). Only one complete pulse is shown in the figure for each of the three cases, i.e., 0.25, 0.1, and 0.05-Hz, at 182 mm/s. However, the power was computed using all four pulses for 0.05 Hz, five pulses for 0.10 Hz, and eight pulses for 0.25 Hz for a fixed velocity to ensure precise evaluation of the expression in Equation (3). In addition, the power of the theoretical case was also computed by noting that the theoretical pressure drop is equal to the effective bed weight, which is shown for the case of 0.05 Hz pulsation in Figure 12a. The shaded area shown in the figure corresponds to $\int_{0}^{T}∆P_{G}dt$ of a single pulse. Therefore, the greater the shaded area, the higher the power or the energy dissipation per unit time. As the frequency increases, the shaded area also increases, thus clearly indicating a greater dissipation of energy.

**Figure 13:**
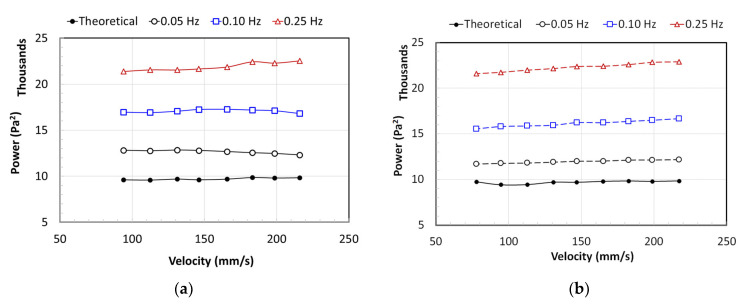
Power evaluated using Equation (3) while (**a**) increasing velocity and (**b**) decreasing velocity.

The power of the overall pressure drop ($∆P_{G}$) signal of a fully fluidized bed is shown in Figure 13. Figure 13a shows the case when the fluidization velocity gradually increased, whereas Figure 13b considers the case of gradual decrease in velocity. The influence of the flow velocity on the magnitude of the power is insignificant. However, as the frequency increased, the energy dissipation due to the pulsation also increased. The theoretical values computed from the effective bed weight lie in the range of 9800 Pa^2^, while those for the 0.05 Hz computed from the experimental data ranged from 11,750 to 12,800 Pa^2^. These values increased to approximately 17,000 and 22,000 Pa^2^ for 0.10 Hz and 0.25 Hz, respectively.

The data are summarized in Table 1, which presents the average values and standard deviations. Clearly, there is an almost 27 ± 4% increase in the energy dissipation when the pulsed flow of 0.05-Hz is used. This increases to 71 ± 5% with a 0.10 Hz and 128 ± 4% with a 0.25 Hz pulsed flow.

## 4. Conclusions

The fluidization of ultrafine and fine powders is often difficult due to the small size of the solid particles, which causes the inter-particle forces to dominate other forces acting on the particles. The fluidization of such powders would reveal poor interphase mixing with severe gas bypassing due to the heterogeneities developed in the bed. Assisted techniques are therefore often required to improve fluidization quality. The efficacy of assisted fluidization techniques has however been mostly evaluated qualitatively in the literature. This mainly consisted of examining the dependence of the pressure drop on the fluidizing gas velocity and evaluating the U_mf_. Any decline in the U_mf_ is considered as an enhancement of the fluidization quality of the powder.

In the present study, we have extensively investigated the fluidization hydrodynamics of ultrafine nanosilica powder, which was subjected to a square-wave pulsed flow of three widely different pulsation frequencies. In addition to the overall pressure drop, the pressure drop characteristics of the lower, middle, and upper portions of the bed were carefully monitored. The dynamics of the upper portion differed from the two lower portions of the bed owing to the bed expansion, which was higher for high-frequency flow pulsations. Moreover, the progress of the collapse process was also felt differently in different portions of the bed when the frequency was changed. For example, the dynamic response of the middle bed region was slowest in comparison to the lower and upper bed regions when incomplete collapse of the bed occurred between two successive pulsation events. These characteristics of the bed dynamics were clearly highlighted by the three- dimensional plot that presented the correlation between the different parts of the pulsed bed. Finally, we have used the signal of the overall pressure drop to compute the power of the signal. A significant improvement in the power was observed when higher frequencies were used. The low-frequency pulsations (0.05 Hz), when compared with unassisted conventional technique, led to an increase of 27%, with further enhancement to 71% and 128%, for a 0.10 Hz and a 0.25 Hz pulsed fluidized bed, respectively.

## Figures and Tables

**Table 1:** Comparison of power input for different pulsation frequencies.

	Theoretical	0.05-Hz	0.10-Hz	0.25-Hz
Mean	9705	12,308	16,588	22,085
Std. Deviation	129	393	545	472

## Data Availability

The data is available on reasonable request from the corresponding author (M.A.).
